# Goat and buffalo milk fat globule membranes exhibit better effects at inducing apoptosis and reduction the viability of HT-29 cells

**DOI:** 10.1038/s41598-019-39546-y

**Published:** 2019-02-22

**Authors:** Xiaoxi Ji, Weili Xu, Jie Cui, Ying Ma, Shaobo Zhou

**Affiliations:** 1 Department of Food Science and Engineering, School of Chemistry and Chemical Engineering, Harbin Institute of Technology, Harbin, 150001 P. R. China; 2 Key Laboratory of Shandong Provincial Education Department: Past-harvest QC and Multiutilization of Characteristic Agricultural Products, Shandong Agriculture and Food Engineering University, Jinan, Shandong 250100 P. R. China; 3 School of Life Sciences, Institute of Biomedical and Environmental Science and Technology, University of Bedfordshire, Luton, LU1 3JU UK

## Abstract

Bovine milk fat globule membrane (MFGM) has shown many health benefits, however, there has not been much study on non-cattle MFGMs. The purpose of this study was to compare the anti-proliferation effects and investigate the mechanisms of MFGMs from bovine, goat, buffalo, yak and camel milk in HT-29 cells. Results showed that protein content in MFGM of yak milk is the highest among five MFGM. All MFGMs reduced cellular viability which was in agreement with cell morphology and apoptosis. However, the number of cells in S-phase from 24 h to 72 h was increased significantly by treatment with goat, buffalo and bovine MFGMs (100 μg/mL), but not yak and camel. All MFGMs treatment significantly reduced the mitochondrial membrane potential (with an order of goat > buffalo > bovine > camel > yak) and Bcl-2 expression, but increased the expression of both Bax and Caspase-3. Taken together, the results indicate that all MFGMs, especially goat and buffalo MFGMs, showed better effects at inducing apoptosis and reduction the viability of HT-29 cells. The mechanism might be arresting the cell cycle at S phase, depolarization of mitochondrial membrane potential, down-regulation of Bcl-2 expression and increase of Bax and Caspase-3 expression.

## Introduction

Milk fat globule membrane (MFGM) is a biopolymer composed primarily of membrane proteins and lipids that surround the fat globules in milk^1^. The concentrations of MFGM in bovine milk are 3.6 g/L of MFGM in cream, with the protein and lipid fractions making up an estimated 22.3% and 71.8%, respectively^2^. MFGM proteins contribute 1–2% of the total protein content in bovine milk, with more than 500 identified proteins^3^. The polar lipids found in MFGM are glycerophospholipids and glycosphingolipids. The complex composition of lipids, proteins and their diverse glycosylation could indicate that MFGM may possess many health-promoting effects^4^: decrease cancer risk^5–7^, cell growth inhibition^8^, anti-bactericidal and anti-inflammatory properties^2,4,9–12^.

Bovine milk accounts for only 42% of consumption in Asia^13^, non-cattle milk (e.g. goat, buffalo, yak, and camel) are consumed more frequently. There is growing interest and importance of understanding the specific functions of such a product category, e.g. modulation of systemic immunity and fecal microbiota^14^; symptom improvement effects in autism^15^ and diabetes^16^, etc. Yak milk contains an enriched level of polyunsaturated fatty acid, particularly conjugated linoleic acid^17^. Buffalo, the second most consumed milk, contains higher content of lipids and proteins^17,18^. The effects of milk on health functions are due to several milk components^13,17^. However, some milk products containing higher proportion of MFGM (e.g., butter milk) are also consumed regularly in these countries^13^. The anticancer activity of bovine buttermilk^19^ and some bioactive peptides of buffalo MFGM were reported^20^, however, there is no report on their anticancer effect, especially MFGMs from non-cattle milk, e.g. yak and buffalo milk.

Colorectal cancer is one of the leading causes of cancer-related death among all types of cancers in the world. Diet plays an important role in generation and prevention of cancer, particularly in relation to the increasing incidence of colorectal cancer^21^. Identifying dietary ingredients or compounds that have antitumour activities may lead to major advances in the prevention of human cancer. Indeed, many natural foods or their bioactivity compounds were shown to possess such pharmacological effects, and have been used or have potential to be used in cancer chemotherapy^22–24^.

In this study, effect of five MFGMs, from yak, bovine, goat, camel and buffalo milk, on the proliferation of human colon cancer HT-29 cells were investigated. The study firstly analysed the compositions of their MFGMs. Then a serial of investigations were carried out to evaluate the effect of the MFGMs on the cell viability, cell cycle, cytomorphology, morphology and microstructure of apoptotic cells, apoptosis, mitochondrial membrane potential (MMP), as well as the expression of Bax, Bcl-2, and Caspase-3 in HT-29 cells. This was the first thorough evaluation of their antiproliferative effect and their mechanisms of apoptosis induction.

## Results and Discussion

### Main compositions of five MFGMs

**Figure 1:**
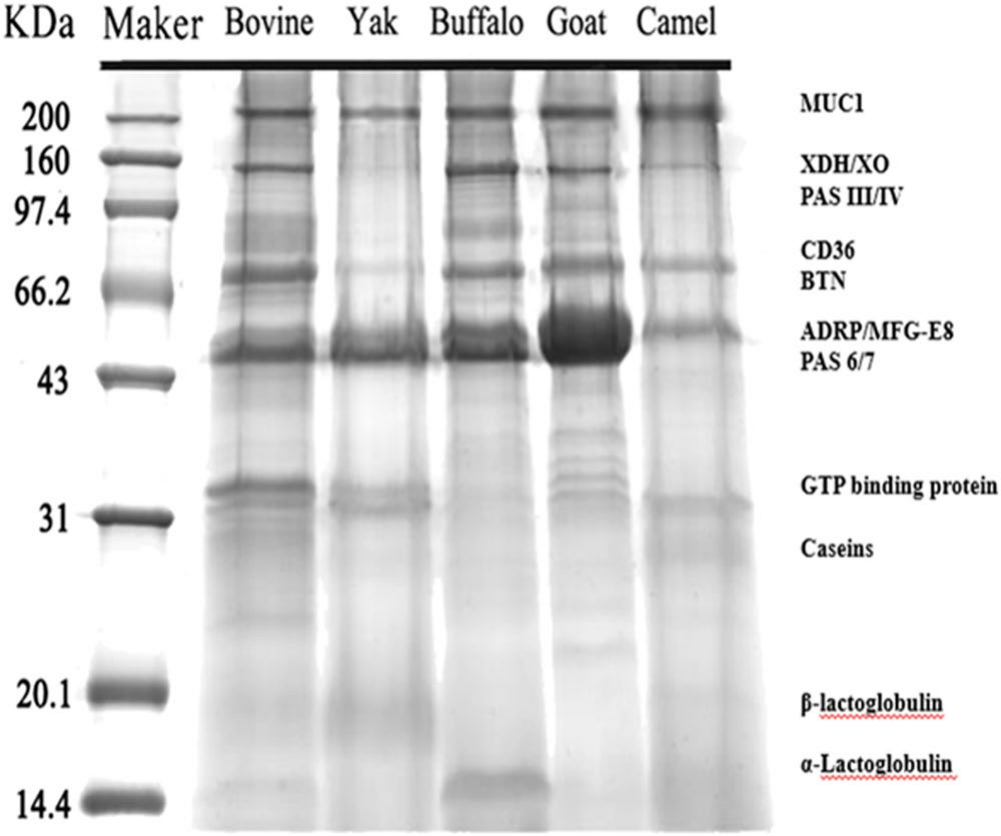
SDS-PAGE of MFGM proteins from bovine, goat, buffalo, yak and camel milk. The main protein bands identified were matched with previous report^28^.

The MFGM contents in five species milk are dramatically different. The order of MFGM content in milk from high to low is, yak milk (0.23%), bovine milk (0.18%), buffalo milk (0.15%), camel milk (0.13%) and goat milk (0.068%). The order of protein contents in MFGM is yak MFGM (425.1 mg/g), buffalo MFGM (416.5 mg/g), bovine MFGM (378.7 mg/g), camel MFGM (348.1 mg/g) and goat MFGM (302.6 mg/g). In our previous research, the chemical compositions of yak and bovine MFGMs^25^ are different depending on methods of isolation, purification and analysis. So far, there is no report on buffalo and camel MFGMs. The different MFGM compositions from different species of milk can be influenced by many factors, such as the size of fat globules, lactation period, and related to the technological treatment as in our previous report^25^. The proteins of the MFGM account only for 1% of the entire globule weight, 25–60% of the membrane weight, and about 1–2% of the total protein weight in bovine milk^3,25,26^. The main protein composition of five MFGMs analysed by SDS-PAGE are shown in Fig. 1, and the named proteins were matched against ours^25,27^ and other studies^25,28^. Most of MFGM proteins aremucin-1 (MUC1, 160~200 kDa), xanthine oxidase [XO, a form of xanthine oxidoreductase (XDH), 146~155 kDa], PAS III (78~98 kDa), cluster of differentiation (CD36, 76~78 kDa), butyrophilin (BTP, 66~67 kDa), adipose differentiation-related protein (ADRP, 50–52 kDa), and milk fat globule-epidermal growth factor 8 (MFG-E8, 46~59 kDa), β-lactoglobulin (20 kDa) as well as α-lactoglobulin (14 kDa).

### Effect of MFGM on cell viability

**Figure 2:**
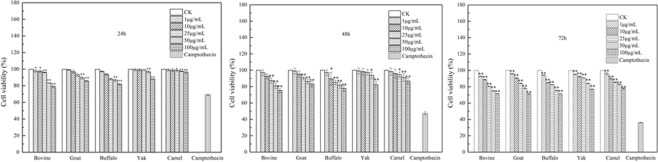
Effect of five MFGMs on HT-29 cell viability by MTT assay. HT-29 cells were treated with each of five MFGMs (1–100 μg/mL), positive control (10 μg/mL, camptothecin) for 24, 48 and 72 h respectively. Data values are expressed as mean ± SD of triplicate determinations. In the same time point and the same concentration, there are significant differences between any two bars labelled with different letters (**a**–**e**) (*p* < 0.05).

HT-29 cells were treated with five MFGM samples (0–100 μg/mL, 0 μg/mL was used as a blank control), and anticancer drug camptothecin as a positive control, for 24, 48 and 72 h respectively. The cell viability were investigated using the MTT assay. Results (Fig. 2) showed that all five MFGMs induced cell death in a concentration-dependent and time-dependent manner, especially at 100 μg/mL and cultured for 72 h, thus they were chosen for the current study. At 72 h, all MFGM samples (100 μg/mL) reduced cell viability, with a relative cell viability rate was 71.32% (bovine), 71.15% (goat), 71.26% (buffalo), 76.85% (yak), 77.92% (camel) and 36.21% (the positive control). Their inhibition of HT-29 cancer cell viability capacities among bovine, goat and buffalo MFGM were the same, but, they were significantly lower than those of yak and camel MFGMs (*p* < 0.05).

### Effect of MFGM on HT-29 cell cycle phase distribution

**Figure 3:**
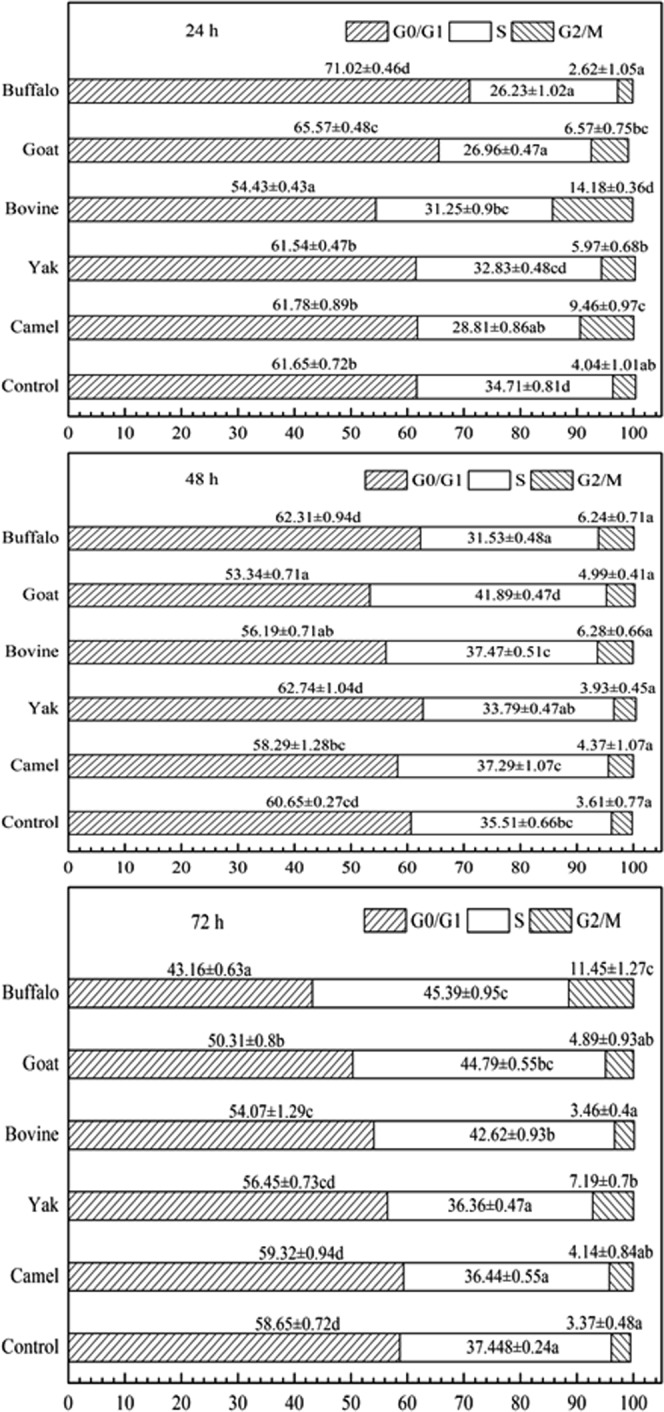
The effect of MFGMs on cell cycle phase distribution of HT-29 cell. Cells were treated with MFGMs (100 μg/mL) for 24, 48, and 72 h respectively. Then the cells were fixed and stained with Propidium Iodide (PI), and the cell cycle was analysed by flow cytometry. Data values are expressed as mean ± SD of triplicate determinations. In the same cellular cycle phase, there are significant differences between any two data sets labelled with different letters (**a**–**d**) (*p* < 0.05).

Anticancer compounds exert their inhibitory effect either by arresting the cell cycle at a particular checkpoint or by induction of apoptosis, or a combined effect of both cycle block and apoptosis^29^. Results from MTT assay revealed that all five MFGMs showed significant antiproliferative activities. In order to further investigate the mechanism of this inhibition, the cell cycle distribution was analysed using flow cytometry. As shown in Fig. 3, when cells were incubated from 24 h to 72 h, treatment with MFGM (100 μg/mL) of bovine, buffalo, goat and camel significantly increased S phase from 31.25% to 42.62%, 26.23% to 45.40%, 26.96% to 44.79% and 28.81% to 36.44%, respectively (*p* < 0.01); but, S phase was not affected by yak MFGM treatment. Treatment with buffalo MFGM, G2 phase increased from 2.62% to 11.45% (*p* < 0.01). Compared with control cell, at 72 h treatment with goat, buffalo or bovine MFGM, G0/G1 phase decreased significantly in cells (*p* < 0.01). These results indicated that S and G0/G1 phase is affected upon addition of bovine, goat and buffalo MGFMs, but not yak at all-time points and camel at 48 and 72 hours.

### Effect of MFGM on cytomorphology

**Figure 4:**
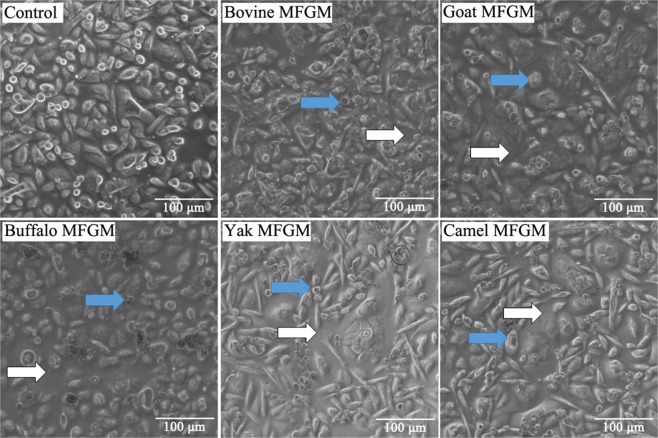
Cytomorphology of HT-29 cells treated with five MFGMs (100 μg/mL) for 72 h. The magnification is ×200. HT-29 cells not treated were used as control cells. Cells with shrinkage () and lost contact between adjacent cells ().

**Figure 5:**
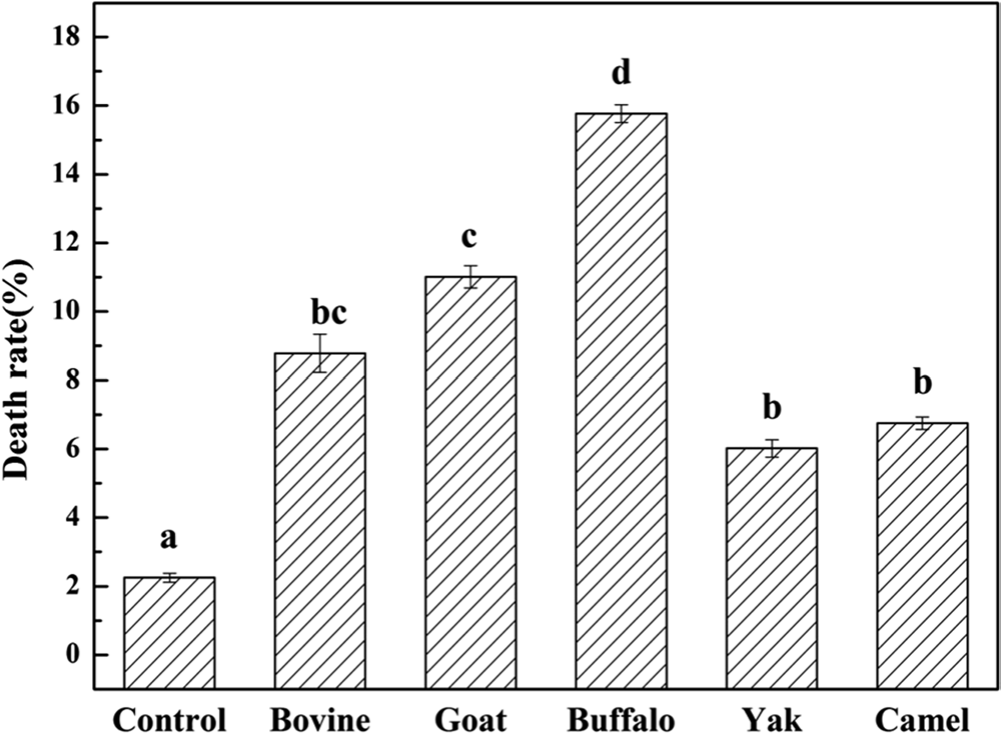
Cytomorphology of HT-29 cells treated with five MFGMs (100 μg/mL) for 72 h. Dead cells including shrinkage and lost contact cells were counted in five view fields. The magnification is ×200. The death rate was calculated by the death/all cells × 100%. There are significant differences between any two bar groups labelled with different letters (**a**–**d**) (*p* < 0.01).

The cytomorphological alterations in HT-29 cells treated with MFGM were observed under an inverted microscope (Fig. 4). The control HT-29 cells distributed evenly on the substratum and attached strongly with adjacent cells; moreover they had transparent cytoplasm, prominent nuclei, as well as a lot of secretions around the cells. However, in HT-29 cells treated with MFGM (100 μg/mL) samples for 72 h, the following were observed: cell shrinkage, decrease in the transparent cytoplasm and nuclear condensation, and lost cellular contact with adjacent cells. Dead cells were counted in five view fields, and the death rate was calculated as the dead cells/all cells ×100%. The order of cell death rate by MFGM was, buffalo > goat > bovine > camel > yak, all significantly higher than control group (*p* < 0.01) (Fig. 5).

### Effect of MFGM on morphology of apoptotic cells

**Figure 6:**
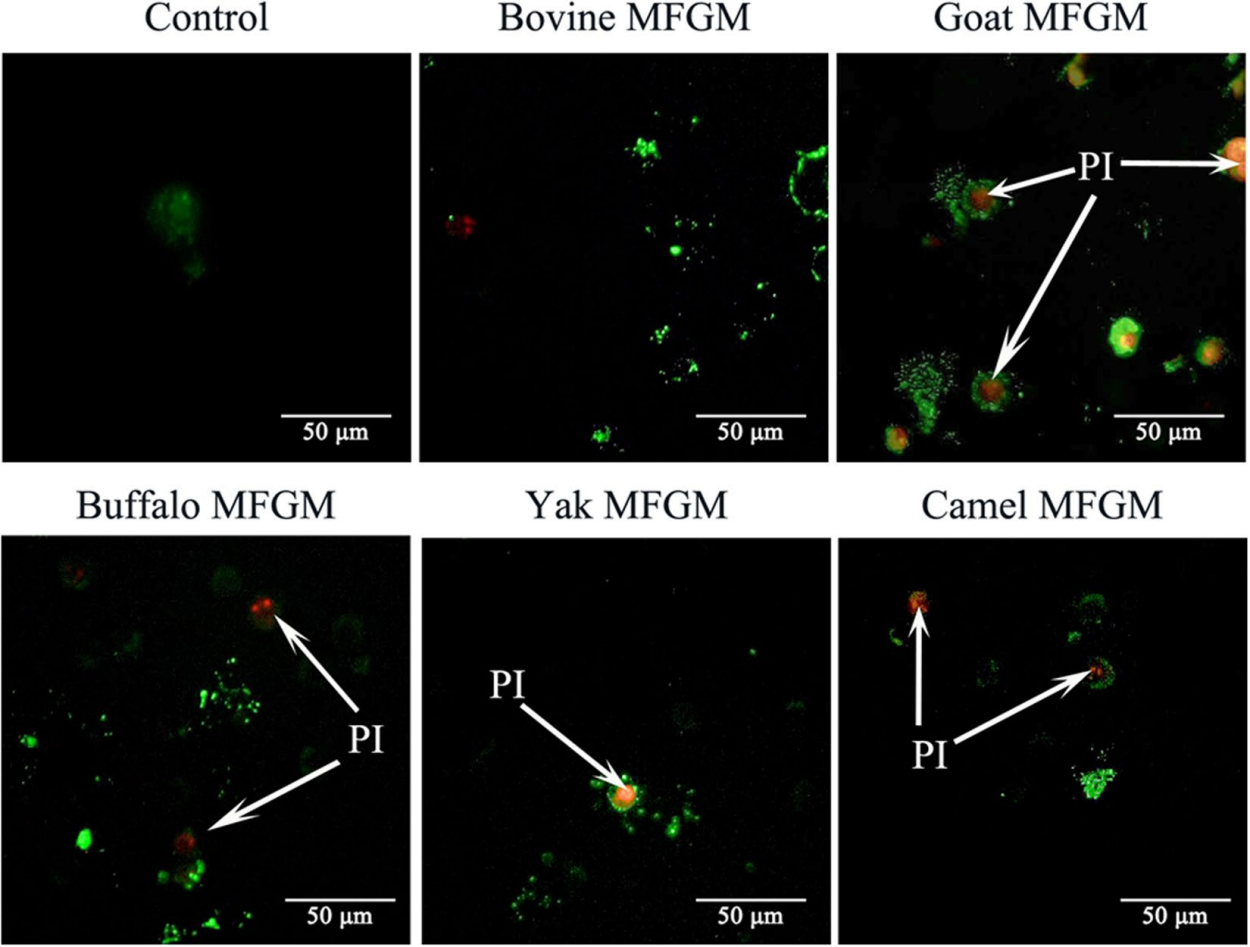
Fluorescence microscopic images of staining annexin V-FITC/PI for apoptotic morphology of HT-29 cells treated with five MFGMs (100 μg/mL) for 72 h. Normal HT-29 cells were used as control cells. Cells stained with V-FITC turn green and indicate they are in the earlier apoptotic phase. While cells stained with both V-FITC (which stains the cell membrane green) and PI (which stains the nuclei red) turn orange, this indicates the cells are at later stages of apoptotic phase. Cells stained with red are only labelled with PI and indicate the dead cells. The magnification is ×200.

**Figure 7:**
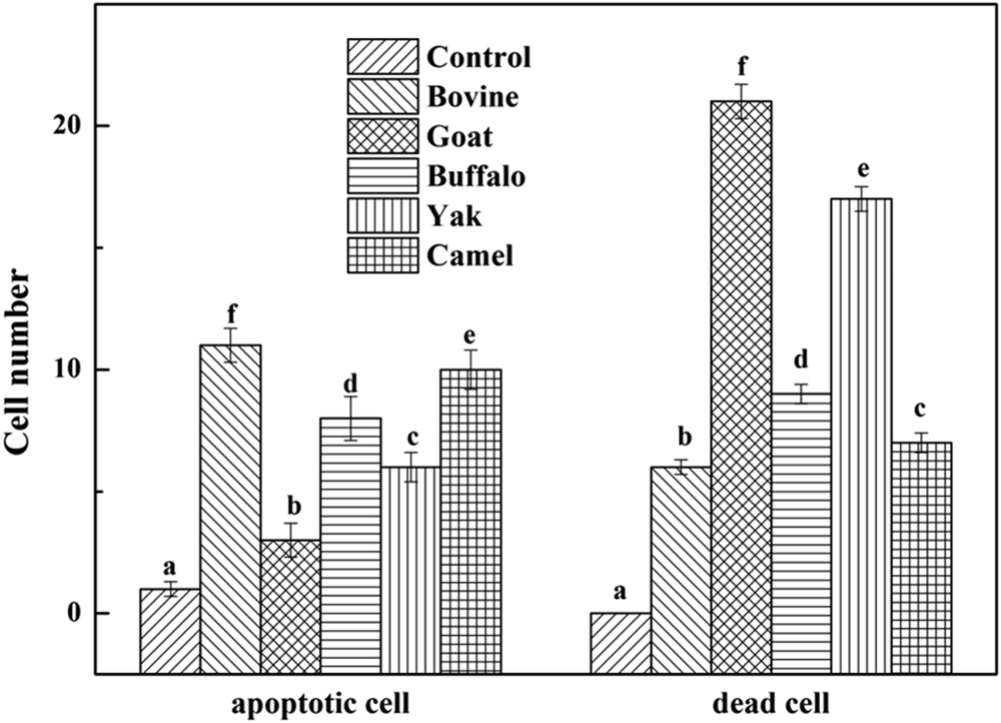
Cell number of apoptotic or dead cells of HT-29 cells treated with five MFGMs (100 μg/mL) for 72 h. There are significant differences between any two bars labelled with different letters (**a**–**f**) (*p* < 0.05).

Cell cycle arrest and apoptosis are the most common causes of cell growth inhibition. Apoptotic morphological changes in HT-29 cells treated with MFGM (100 μg/mL) for 72 h, were assessed by using annexin V-FITC/PI staining assay. In this assay, treated cells were double stained by annexin V-FITC and PI, then examined by fluorescence microscopy to identify the distribution of early (stained as green with V-FITC but not PI, V-FITC+/PI−) and late (stained as orange, as it was merged colours of green and red V-FITC+/PI+) apoptotic cells and necrotic cells (stained only red, V-FITC−/PI+) (Fig. 6). A normal intact nuclear architecture emitted green fluorescence in cells with normal viability in untreated control cells. On the other hand, characteristic apoptotic morphological characteristics were found in cells treated with MFGMs. These included viable cells with condensed nuclei, chromatin condensation, irregular round shape, fragmented structure as well as non-viable cells with apoptotic nuclei, and some apoptotic bodies^30^. These results indicated that the cytotoxic effect of the MFGMs in cells was mediated through apoptosis. Among five MFGMs treatment, the average total apoptotic cell number including cells in the earlier and late stage of apoptotic phase (Fig. 7) under five view fields was showed in the following order, buffalo > goat > bovine > camel > yak > control. These results were different from MTT assay, as well as cytomorphology changes observed under inverted microscope. The differences between MTT and morphology results could be caused by cell type, density of phosphatidyl serine (PS) on the cell membrane, ratio of PS externalization in apoptotic cells, and methods of inducing cell apoptosis^31^. Annexin V-FITC/PI double staining is a more sensitive method in detecting apoptosis^32^. Externalization of PS from the inner side to outer leaflet of the cell membrane is an important indication of early apoptosis^33^. Because annexin V-FITC possesses a high affinity towards PS, early apoptotic cells can be easily detected by fluorescently labelled annexin V-FITC. Meanwhile, PI can detect necrotic cells due to its permeability through the damaged cell membranes^34^. Among five MFGMs treatment, the average dead cell number under five view fields was showed in the following order, buffalo > goat > bovine > camel > yak > control. When adding the apoptotic cells and dead cells together, the order of the total number in five MFGMs treatment showed the same effect which is in the same trend, buffalo > goat > bovine > camel > yak > control.

### Effect of MFGM on microstructure of apoptotic cells

**Figure 8:**
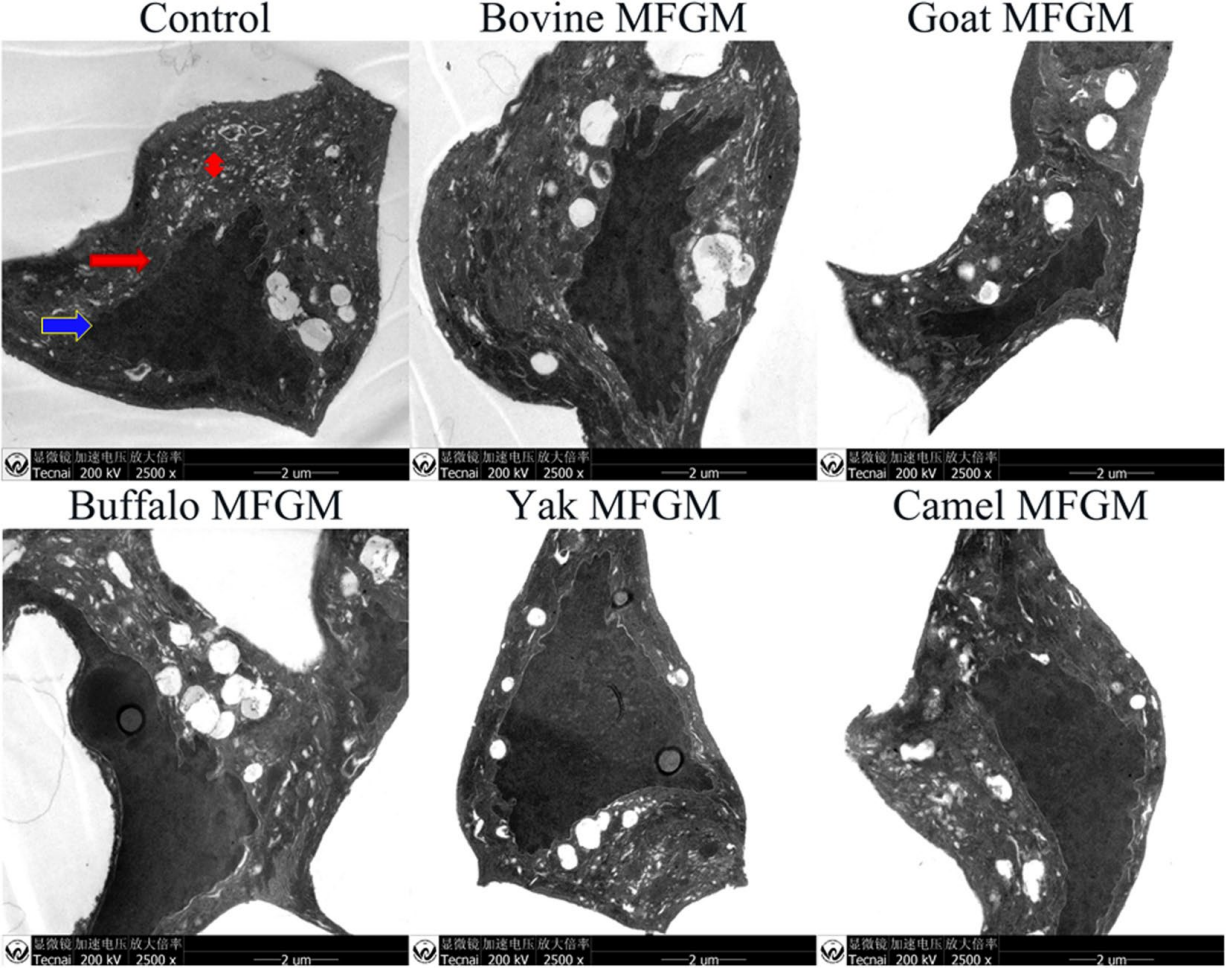
Nuclear changes of apoptotic HT-29 cells treated with MFGM (100 µg/mL) for 72 h. HT-29 cells without given treatment were used as control cells. Images of TEM, The magnifications is ×2500. Cell membrane (); nuclei (); cytoplasm ().

The microstructure of apoptotic cells was examined with transmission electron microscopy (TEM). The TEM micrographs of apoptotic HT-29 cells treated with MFGM (100 μg/mL) for 72 h are shown in Fig. 8. HT-29 cells without treatment have intact and smooth cellular membrane and nuclear membrane, and the surface of the cells are covered with microvilli. In cells treated with MFGM, chromatin was grouped together in clear masses, vacuoles distributed in the cytoplasm which became sparse, and organelle structure became vague^35,36^. Among cells treated with five MFGMs, early apoptotic cells, with features of mitochondrial hyperplasia, increased lysosomes and no obvious pyknotic nuclei, were observed in cells treated with bovine and buffalo MFGM. However, late apoptotic cells were seen in cells treated with yak MFGM, the features of late apoptotic cells were homogeneous karyopycnosis and a large number of vacuolated cell in the cytoplasm, disappearance of lysosomes, and many granular residues. The above features indicated that apoptotic processes was speeded up in cells after MFGM treatment.

### Effect of MFGM on apoptosis analysed by flow cytometry

**Figure 9:**
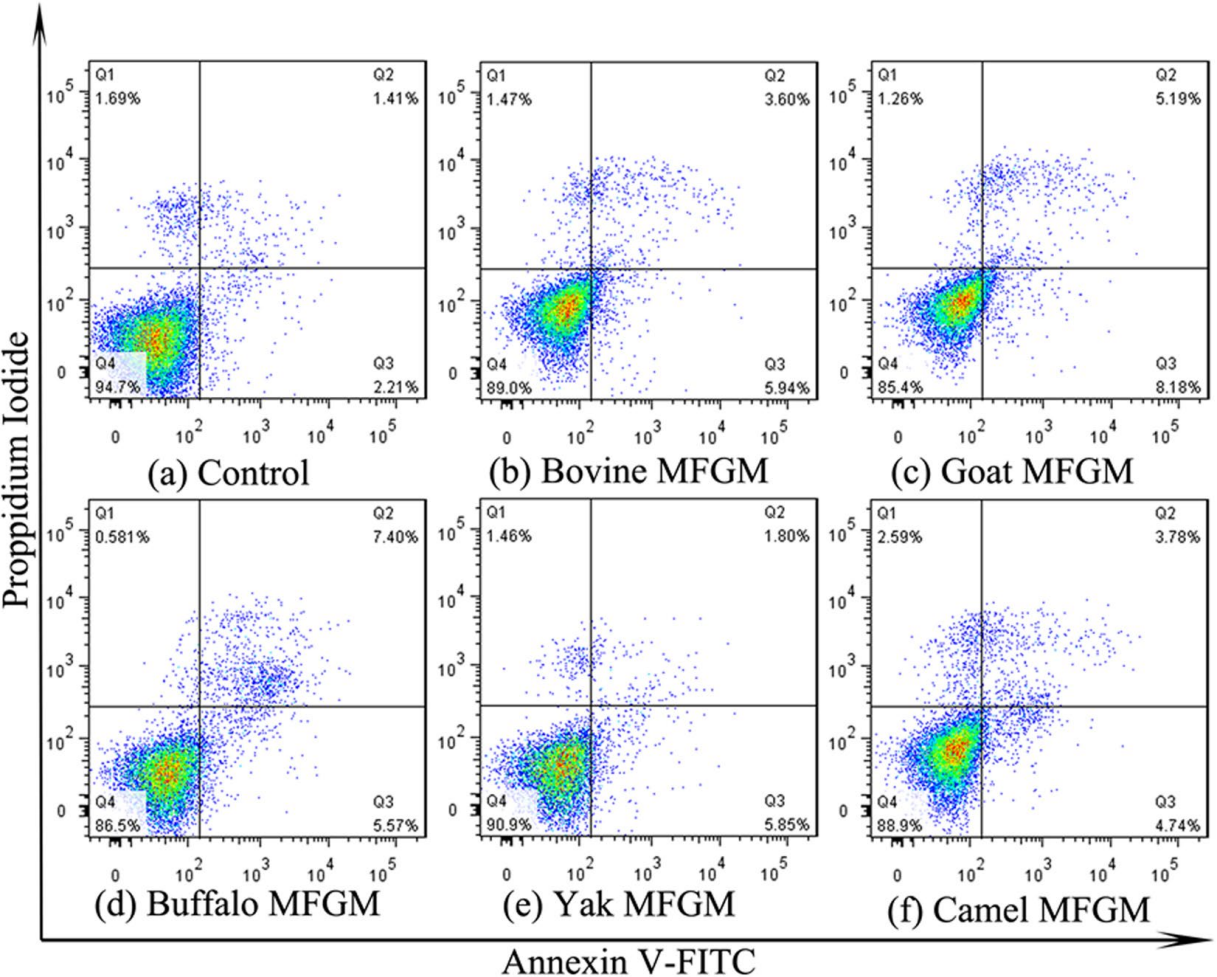
Effect of MFGMs on apoptosis by flow cytometry. HT-29 cells without given treatment (Control group) or treated with each of five MFGMs (100 µg/mL) were labelled with annexin-V- FITC (V-FITC) and PI.

**Figure 10:**
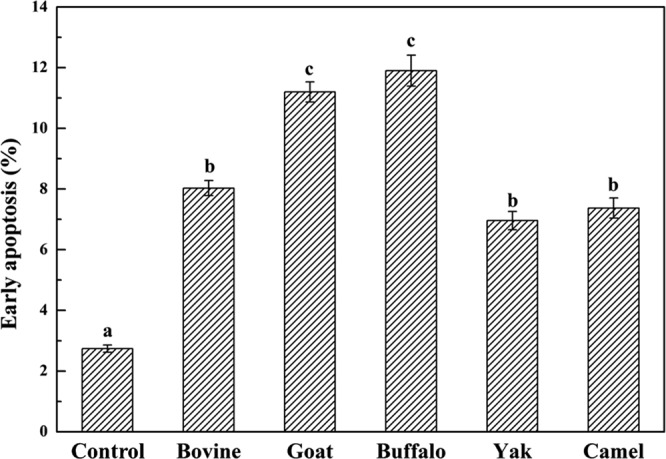
The early apoptotic rate of HT-29 cells induced by MFGMs was analysed with the flow cytometer. Data values are expressed as mean ± SD of triplicate determinations. There are significant differences between any two bars labelled with different letters (**a**–**c**) (*p* < 0.05).

HT-29 cells were treated with five MFGMs (100 μg/mL) respectively, then stained with annexin V-FITC/PI and analysed by flow cytometry. Apoptotic cells significantly increased in cells treated with MFGM within 72 h (Figs 9 and 10). The order of higher to lower apoptosis cell rate was buffalo > goat > bovine > camel > yak. MFGM significantly attenuated cells in early apoptosis compared with control (*p* < 0.01), the early apoptotic rate was about 11.9% in buffalo MFGM and 11.2% in goat MFGM which were significantly higher than 8.03% in bovine MFGM,7.37% in camel MFGM and 6.96% in yak MFGM. The results indicated that buffalo and goat MFGM were more effective in inducing apoptosis than other three MFGMs. The results were consistent with the previous observations of cellular morphology. The results indicated that MFGM could effectively reduce cell growth of HT-29 cells and induced apoptosis *in vitro*.

### Effect of MFGM on mitochondrial membrane potential (MMP)

Mitochondria are important organelles that are involved to release of apoptotic signals via an intrinsic pathway for the execution of apoptosis^37^. Dysfunction of the mitochondria leads to the reduction of MMP and leads to the release of cytochrome C from the mitochondria into the cytosol^38^. Depolarization of MMP is an early characteristic of apoptosis. MMPs of HT-29 cells treated with MFGM (100 μg/mL) was analysed using flow cytometry. As shown in Table 1, the MMP values of all groups treated with MFGM, were significantly lower than that of the control group (*p* < 0.05). The results indicated that all of five MFGMs significantly reduced the MMP of HT-29 cells, with an order of goat > buffalo > bovine > camel > yak.

**Table 1:** Effect of MFGMs on mitochondrial membrane potential (MMP) of HT-29 cells.

	MMP (mV)
Control	184.61 ± 9.21
Bovine MFGM	113.34 ± 8.64*
Goat MFGM	84.55 ± 12.36*
Buffalo MFGM	96.47 ± 8.47*
Yak MFGM	165.72 ± 10.62*
Camel MFGM	144.75 ± 11.23*

Note: HT-29 cells were treated with each of five MFGMs (100 μg/mL) for 72 h. Data values are expressed as mean ± SD of triplicate determinations. *p < 0.01 as compared with control group.

**Figure 11:**
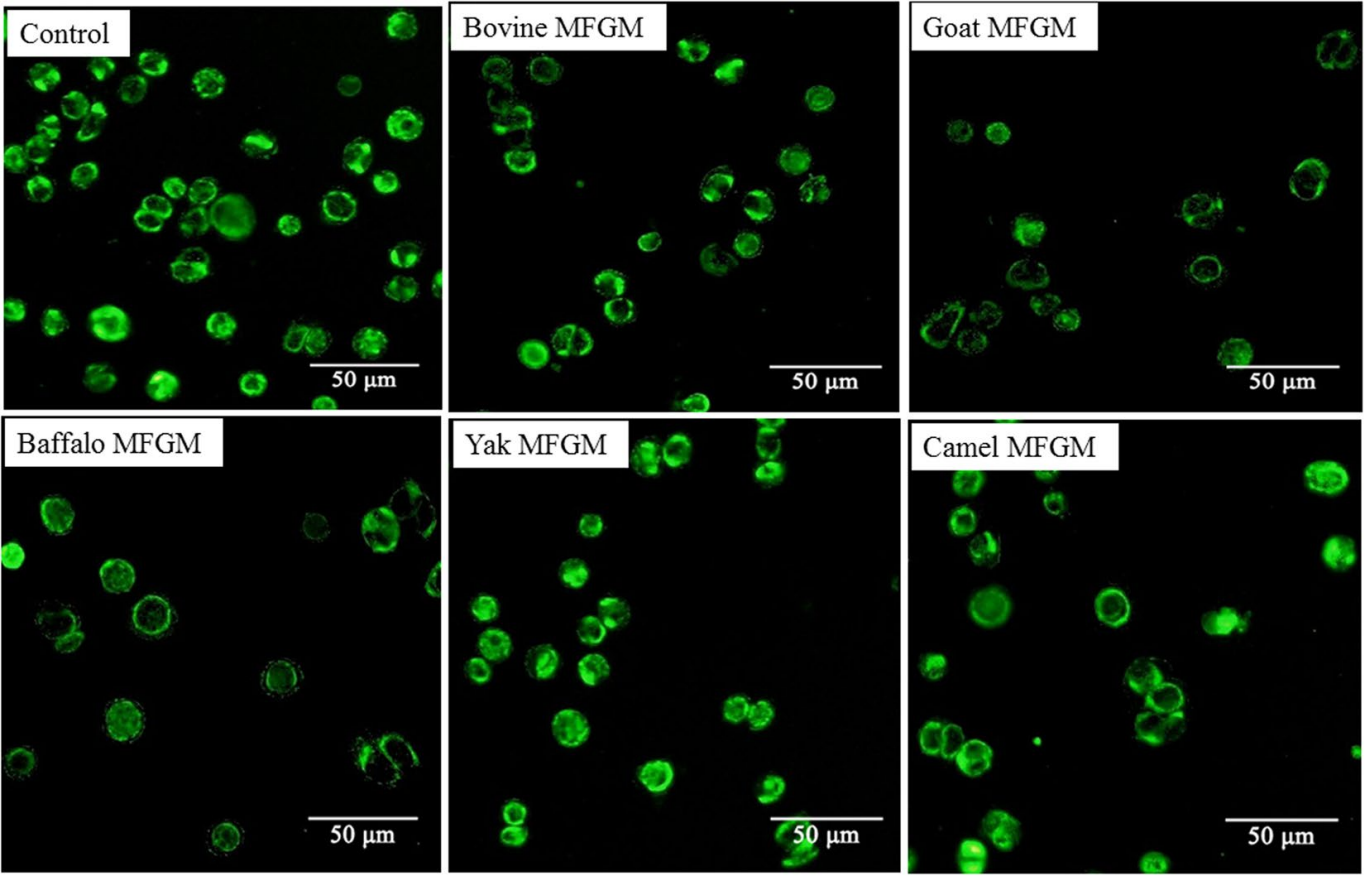
The mitochondrial content was labelled with a mitochondrial green fluorescent probe-Mito-Tracker Green in HT-29 cells treated with MFGMs (100 μg/mL) for 72 h and visualized with a fluorescence microscope.

**Figure 12:**
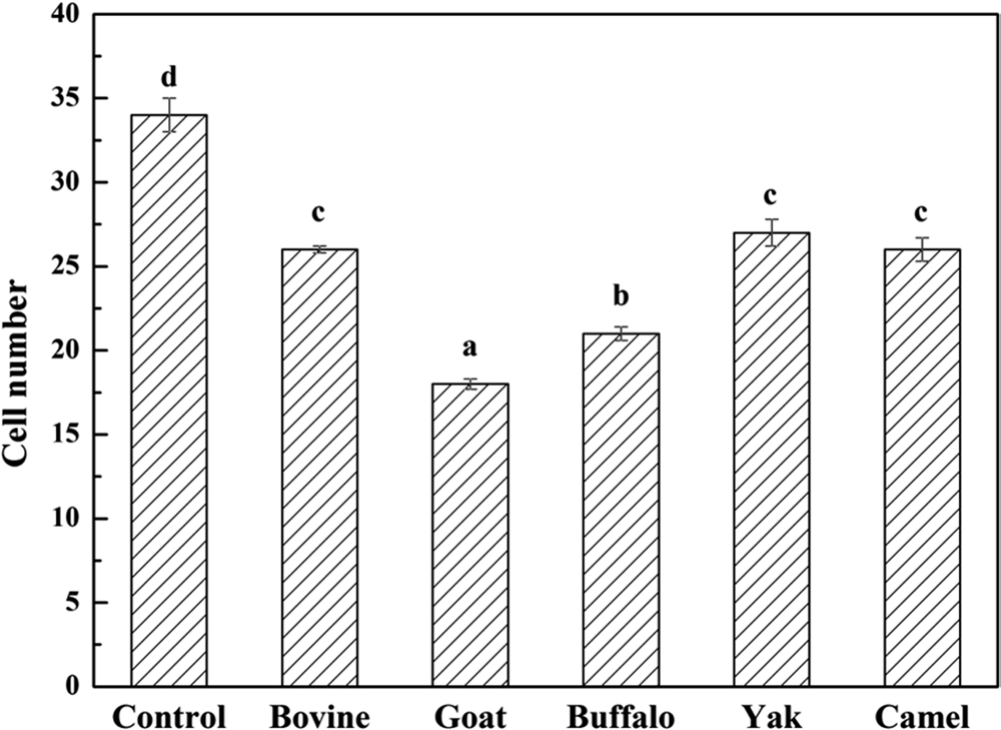
Cells labelled with green are analysed. Data values are expressed as mean ± SD. Between any two bars labelled with different letters, *p* < 0.05.

The mitochondrial content was labelled with a mitochondrial green fluorescent probe-Mito-Tracker Green in HT-29 cells treated with MFGM and visualized with a fluorescence microscope (Fig. 11). Under five view fields, the order of average cells labelled with green in each field is, control > yak > camel > bovine > buffalo > goat (Fig. 12). The results further indicated that MFGM could induce apoptosis by disrupting mitochondrial function induced by a reduction of MMP.

In summary, the above results showed that all MFGM samples (100 μg/mL at 72 h) significantly: (1). reduced HT-29 cancer cell viability, especially by goat, buffalo and bovine MFGM (about 28%), significantly higher than yak and camel MFGM (22–23%); (2). affected the cell cycle, showed strong cell cycle arrest, via increasing the S phase in goat, buffalo and bovine MFGM (45.4–42.6%) which all significantly higher than both yak and camel MFGM (36.4%), as well as increasing G2/M phase in buffalo (11.5%), in yak (7.9%) and goat MFGM (4.9%) groups, but not in bovine and camel MFGM groups; (3). The cytomorphology study also showed the order of cell death rate was, buffalo > goat > bovine > camel > yak, all significantly higher than the control group; (4). Effect on morphology, the order of the total number of the apoptotic cell and dead cell in five MFGM treatments was, goat > yak > buffalo > camel > bovine > control; (5). Apoptosis analysis by flow cytometry, the order of higher to lower apoptosis cell rate was buffalo > goat > bovine > camel > yak; (6). All MFGMs significantly reduced the cells MMP, with an order of goat > buffalo > bovine > camel > yak; as well as (7), the intracellular fluorescence intensity with an order of control > yak > camel > bovine > buffalo > goat. All these results indicated that all MFGMs reduced cell viability, this may be caused by inducing cell apoptosis, a reduction of MMP which may disrupt mitochondrial morphology and function. Among all tested MFGMs, buffalo and goat MFGM were more effective in inducing apoptosis than other three MFGMs.

In our previous study, using iTRAQ techniques, 424 proteins were identified with 146 proteins significantly different between bovine and buffalo MFGMs^27^. These include enzymes, immunoglobulins, and proteins from the secretory epithelial cell cytoplasm, leukocytes and skim milk components. Even more, the contents of sodium phosphate solute carrier protein and gama-glutamyl transpeptidase are significantly higher in buffalo MFGM, they possess carriers and catalytic activities, and serve as the basic composition of biosynthesis and metabolism^38^. There were 336 proteins further identified by iTRAQ technology among all five MFGMs. In Fig. 1, several proteins were matched, among them several proteins showed antioxidants effect and affect cancer cell growth. MUC1 participates in intracellular signal transduction pathways^39^. Its overexpression was found in the pathogenesis of papillary thyroid carcinoma^40^ and plays an immunoprotective role in inflammation conditions^41^. XO is an enzyme that generates reactive oxygen species^42^. It can inhibit the growth of bacteria by increasing hydrogen peroxide formation^43^. PAS III is a cell membrane-associated mucin-like glycosylated protein^44^. Its overexpression was also found in papillary thyroid carcinoma^40^. CD36 is also known as platelet glycoprotein 4, fatty acid translocase and scavenger receptor class B member 3. CD36 plays a role in the regulation of angiogenesis, fatty acid uptake which may promote cancer cell migration and proliferation in hepatocellular carcinoma, glioblastoma^45,46^, and hepatocellular carcinoma^47^. BTP belongs to the immunoglobulin superfamily showed negative regulation of lymphocyte activation as well as to be associated with autoimmune diseases and cancer cell inhibition^48^. The highest density band of ADRP and MFG-E8 was expressed in goat, buffalo, yak and bovine, but less in camel MFGM. The amount of ADRP in goat was consistent to Cebo’s^23^ and Spertino’s results^49^. ADRP was rapidly induced during adipocyte differentiation, its expression levels in the primary tumours were measured, whereas it was down-regulated in undifferentiated tumours^50^. MFG-E8 is highly glycosylated *in vivo* and plays a role in inflammatory responses and inflammatory/autoimmune diseases^51^. The differing compositions of the bioactive proteins of MFGMs might contribute to the inhibition of HT-29 cancer cells’ growth. It will be worthwhile to further investigate which specific component plays the inhibitory role.

### Effect of MFGM on expression of Bax, Bcl-2 and Caspase-3

**Figure 13:**
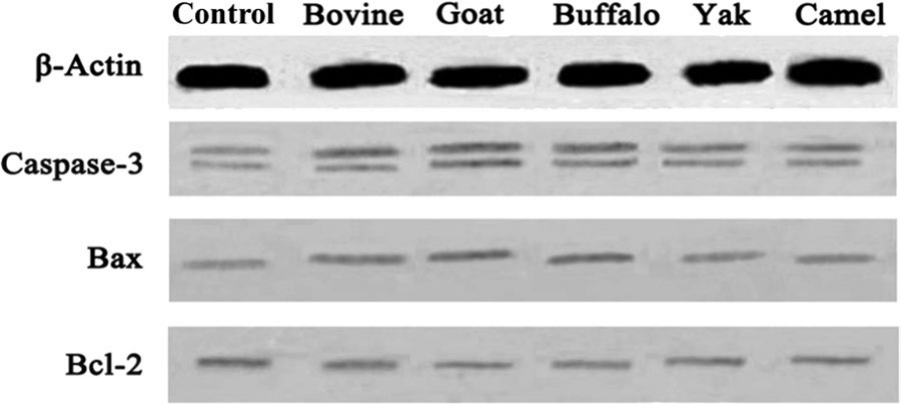
Effect of each of five MFGMs on the expressions of Caspase-3 (19 kDa and 17 kD), Bax (21 kDa), and Bcl-2 (26 kDa) were analysed through *Western blot* assay.

**Figure 14:**
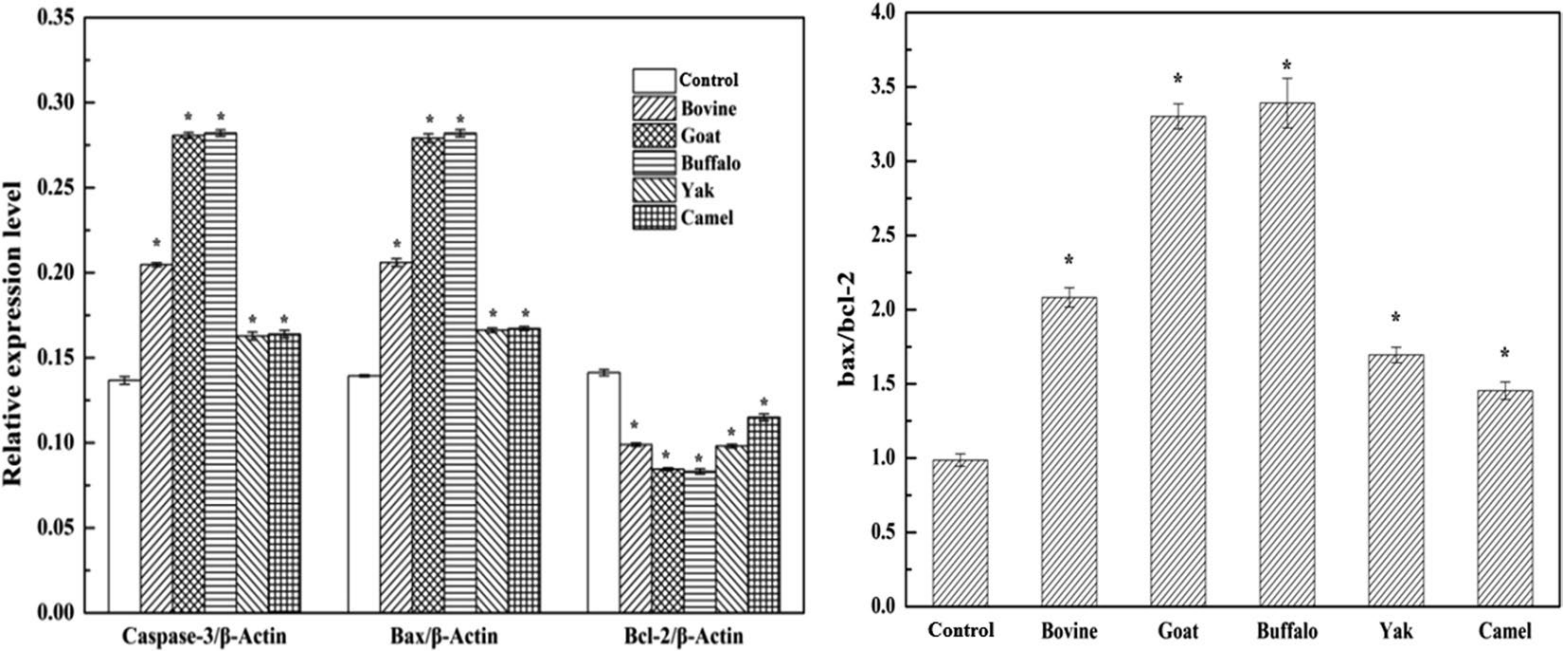
Effect of each of five MFGMs on the expressions of Caspase-3, Bax, and Bcl-2 was calculated by the comparison to β-Actin (42 kDa) (Left). The ratio of Bax/Bcl-2 was calculated as apoptosis index (Right). Data values are expressed as mean ± SD of triplicate determinations. *p < 0.01 as compared with control group.

Apoptosis is a complex process and is regulated by a variety of factors^52,53^. Among the factors, two groups of proteins are involved in apoptotic cell death, they are members of the Bcl-2 family^54^ and a class of cysteine proteases known as caspases^55^. The Bcl-2 family proteins regulate apoptosis by controlling the mitochondrial membrane permeability^56^ through stabilizing the mitochondrial membrane, while Bax induces apoptosis by enhancing mitochondrial membrane permeability, which leads to the release of cytochrome C from mitochondria^57^. Activation of Caspase-3 is regarded as a primary mechanism of apoptosis^55,58^. Caspase-3 can be activated through cytosolic release of cytochrome C by Bax protein^59^. In one study, Caspase-3 activity was increased in SGC-7901 cells treated with MFGM^60^. To gain better insight into the mechanisms underlying MFGM mediated apoptosis, the effects of five MFGM on the expression of major pro-apoptotic proteins were tested after cells were exposed to individual MFGM (100 μg/mL) for 72 h (Fig. 13). The expression of Bcl-2 in cells of all five MFGM groups was decreased significantly, moreover, there was a significant decrease in buffalo and goat MFGM compared to that of other three groups (Fig. 14, left) (*p* < 0.01). Compared with the control group, the level of both Bax and Caspase-3 as well as the ratio of Bax/Bcl-2 (Fig. 14, right) in HT-29 cells increased after treatment with all five MFGMs. There was a significant increase in buffalo and goat MFGM compared to that of bovine, yak and camel MFGM (*p* < 0.01). These results revealed that all five MFGMs induced apoptosis in HT-29 cells by decreasing Bcl-2 expression and increasing Bax and Caspase-3 levels. And among them, buffalo and goat MFGMs showed the strongest effect.

In conclusion, the results demonstrated that all five MFGMs, from bovine, buffalo, camel, yak and goat milk, reduced the viability of human colon carcinoma HT-29 cells, which was in agreement with cellular morphological changes. MFGM, a mixture biopolymer, contains a variety of proteins, phospholipids, fatty acids as well as trace elements among different sources^14^. This would be the cause of their different responses on inhibition of HT-29 cell growth. Among them, goat, buffalo and bovine MFGMs, showed better effect. This may be caused by inducing apoptosis, by arresting the cell cycle in S phase, decreasing mitochondrial membrane potential, down-regulating Bcl-2 expression and increasing Bax, and cleaved-caspase-3 expression levels. The data suggests that five MFGMs, especially the new discovery of goat and buffalo MFGMs, might be potential agents for the prevention of human colon cancer.

## Methods

### Isolation of MFGM

All five species milks were collected from a milk tank of individual dairy farm, which containing a mixture milk of 50–80 lactating animals in a variety of lactation stages. Bovine and goat milk were collected from Jiangbei Dairy Farm in Harbin, Heilongjiang Province; Yak milk was collected from Hongyuan County Dairy Farm, Abei Zhou, Sichuan Province; Camel milk was collected from a Xilin Guolemeng Shuni Teguqi Dairy Farm, Inner Mongolia and buffalo milk was from Dairy Farm of Institute of Buffalo Research Institute, Guangxi Provinces, China. The milk were stored at 4 °C and shipped into the lab. The MFGM samples were isolated within 24 h from milking using the following procedure. Cream was separated by centrifugation at 3500 × *g* (4 °C, for 30 min) (TDZ4B-WS, Shanghai, China). The cream was resuspended in 10 mM phosphate buffer (pH 7.5) at a ratio of 1:1 (cream:buffer) at 40 °C and stirred slowly for 10 min. Next it was centrifuged at 4000 × *g* (25 °C, for 15 min) to remove serum constituents. The remaining cream was washed twice with deionized water to remove residual phosphate buffer. The same volume deionized water was added into the cream, and the mixture was slowly stirred until cream totally melted in a 50 °C water bath. Fat globule disruption was achieved by an ultrasonic cell disrupter (20 Hz, 10 s), and then it was centrifuged to remove fat at 15000 × *g* at 25 °C for 20 min. Following freeze-drying, the MFGM pellets were obtained and kept at −20 °C^61^. MFGM from same preparation were used for all the experiments.

### Analysis of MFGM compositions

MFGM content of whole milk was calculated by the weight of dried MFGM divided by the weight of the whole milk. The protein content of MFGM was determined by the Dumas combustion method via measuring total nitrogen and multiplying by a factor of 6.38^62^. The different protein components of MFGM, 10 mg of each sample were loaded into a12.5% sodium dodecylsulfate polyacrylamide gel (SDS-PAGE) under 100 V, for 70 mins. The gels were stained with Coomassie blue R-250 for 1 hour and distained with a solution of methanol, water, acetic acid (5:4:1, v-v:v), and the gel bands were matched to a previously published literature^63^.

### Cell culture and cell viability assay

The human colon cancer cell line, HT-29 cells (Shanghai Cell Bank, China) were cultured in RPMI-1640 medium (Sigma, USA) at 37 °C in 5% CO_2_/95% air. Cell culture medium was replaced every 24 h until the highest cell density was reached. Cell viability was determined by 3-(4,5-dimethylthiazol-2-yl)-2,5-diphenyltetrazoliumbromide (MTT) assay which assesses the total metabolic activity/cell viability, a mixture of cell proliferation rate, cell size, metabolic rate and cell survival^63,64^. The cancer cells were seeded at a concentration of 5 × 10^4^ cells/mL in 96-well tissue culture plates and total volume was adjusted to 100 μL with growth medium cultured at 37 °C in 5% CO_2_/95% air for 24 h. The cells were then cultured in medium with various concentrations (0, 1, 10, 25, 50, 100 μg/mL), in direct comparison to a study on bovine MFGM^60^ at (24 h, 48 h, and 72 h). Five MFGM samples were solubilized in RPMI1640 medium (Sigma, USA). Camptothecin (10 μg/mL, Sigma) was used as a positive control, as it is a well-known apoptotic inducer at 4–10 mg/ml and it has been used in different tumour cells^65,66^. After treatment, 15 μL MTT of 0.5 mg/mL was added into the medium, and incubated at 37 °C in 5% CO_2_/95% air for further 3 h. The medium was replaced with 200 μL dimethyl sulfoxidethen then they were gently shaken for 10 min at room temperature. Absorbance was measured at 492 nm with a Microplate Reader (Thermo MK3, Thermo Fisher Scientific, USA). Cell viability rate (%) was calculated as in the following formula: 100% − [OD_blank (Absorbance of cells without given MFGM treatment)_ − OD_sample Absorbance of cells given with MFGM treatment_]/OD _blank_ × 100%.

### Analysis of the cell cycle

The cell cycle was measured by DNA fragment staining with PI. HT-29 cells (1 × 10^5^ cells/well) seeded in a 24 well plate were treated with MGFM from five species at 100 μg/mL, which was based on the results of MTT assay, in 37 °C, 5% CO_2_ for 24 h, 48 h and 72 h. The cells were collected following mild trypsinization, and then centrifuged at 1000 × *g* for 3 min. The collected cells were washed twice with PBS and fixed with cold ethanol (70%) for 24 h at 4 °C. After fixation, DNA fragments were stained in PBS (50 μg/mL PI and 100 μg/mL RNase) for 30 min at 7 °C. The cells were washed with cold PBS and centrifuged at 1000 × *g* for 3 min. The fluorescence intensities of viable cells were measured using a flow cytometer (BD Accuri C6, USA).

### Morphologic observation of HT-29 cells

HT-29 cells (1 × 10^5^ cells/well) were seeded into a six well plate and incubated at 37 °C in 5% CO_2_/95% air for 24 h. Five MFGM samples were added into the six well plate to a final concentration up to 100 μg/mL for each MFGM, and then the cells were further incubated for 72 h at 37 °C in 5%CO_2_/95% air. Following incubation, the cytomorphology of cells was examined under an inverted microscope (Leica DMI3000B, Germany) at ×200 magnification.

Above treated cells were washed twice with 0.1 M PBS (4 °C). The cells were covered with 300 µL 1 × binding buffer, and then mixed with 5 µL annexin V-FITC (Shanghai Beibo Ltd, China). After 15 min of incubation in the dark, cells were stained with PI (10 µL, 50 g/mL), and incubated in the dark for 10 min. Following incubation, cells were washed with PBS (twice, 5 min each) and examined for condensed/fragmented nuclei under a fluorescence microscope (Leica IX71, Germany) at ×200 magnification. The transmission electron microscopy (TEM) of apoptotic HT-29 cells was performed as described previously^67^. HT-29 cells were respectively treated with each of five MFGM samples (100 μg/mL) for 72 h at 37 °C in 5% CO_2_/95% air. The cells were trypsinized and centrifuged at 1000 × *g* (3 min) to remove the medium. The harvested cells were fixed in 2.5% glutaraldehyde for 2 h, and rinsed several times with 0.1 M PBS. The cells were fixed again with 1% osmium tetroxide for 1 h, then rinsed three times with 0.1 M PBS, and further dehydrated with ethanol. After that, they were, embedded in Epon812 epoxy resin and polymerized at 60 °C for 48 h. The samples were sectioned on a Leica UC6 microtome to 70 nm thickness, collected on 300 mesh copper grids, and counterstained with uranyl acetate and lead citrate. Imaging was done with a Tecnai 12 transmission electron microscope (FEI, USA) at an accelerating voltage of 200 kV.

### Determination of apoptosis by flow cytometry

The cultured HT-29 cells (1 × 10^5^ cells/well) were seeded into a six well plate and treated with each of the five MFGM samples at 100 μg/mL for 72 h at 37 °C, 5% CO_2_. The cells were collected following mild trypsinization, and then centrifuged at 1500 × *g* for 5 min. The trypsinized cells were washed with PBS, and resuspended in 300 μL of 1 × binding buffer, and mixed with 5 μL of FITC-conjugated annexin V and 10 μL PI. They were then incubated at room temperature in the dark for 15 min. Labelled cells were analysed using a flow cytometer (BD Accuri C6, USA).

### Determination of mitochondrial membrane potential (MMP, mV)

HT-29 cells (1 × 10^5^ cells/well) seeded in a 24 well plate were treated with each of the five MFGM samples at 100 μg/mL for 72 h at 37 °C, 5% CO_2_. The cells were washed with PBS first, then stained with Mito-Tracker Green followed by incubation for 30 min at 37 °C. The excess dye was removed by washing with PBS and the cells were then observed under the fluorescence microscope at ×200 magnification. After incubation, the MMP values in HT-29 cells were analysed using a flow cytometer (BD Accuri C6, USA).

### *Western blot* analysis

The cultured HT-29 cells were treated with MFGM from five species of milk at 100 μg/mL for 72 h at 37 °C, 5%CO_2_. The HT-29 cells were lysed in RIPA lysis buffer (containing PMSF) for 2 h, then they were sonicated and centrifuged for 10 min at 4 °C at 12000 × *g*. Protein concentration in the cell lysates was determined by Lowry’s method^68^. Proteins were separated by 10% SDS–PAGE, and transferred onto a PVDF membrane. Membranes were washed with TBST (10 mM Tris, 100 mM NaCl, 0.1% Tween 20) followed by blocking with 5% skim milk powder in TBST for 1 h at room temperature. The membranes were incubated overnight (4 °C) with specific primary antibodies (Bax, Bcl-2, Caspase-3, and β-Actin) (Santa Cruz Biotechnology, Santa Cruz, CA, USA). Membranes were washed twice with TBST, incubated with appropriate secondary antibody conjugated with HRP for 2 h at 37 °C, and the bands were visualized with an ECL chemiluminescence kit (Millipore Corporation, Billerica, MA, USA). Image J software was used to analyze gray-scale value of bands.

### Statistical analysis

Statistical analysis was performed on all the relevant experiments relative to controls using SPSS software (version 20). ANOVA followed by the Tukey’s test was used to analyze the statistical significance between groups. Significance was set at *p* < 0.05.
